# Factors associated with unintentional weight loss among older adults in a geriatric outpatient clinic of university hospital

**DOI:** 10.1371/journal.pone.0260233

**Published:** 2021-11-18

**Authors:** Chuthamas Sripongpunkul, Aisawan Petchlorlian, Tanchanok Chattaris, Saran Thanapluetiwong, Orapitchaya Sriwannopas, Sirintorn Chansirikarnjana, Taweevat Assavapokee, Praopilad Srisuwarn, Sirasa Ruangritchankul

**Affiliations:** 1 Division of Geriatric Medicine, Department of Medicine, Faculty of Medicine, Ramathibodi Hospital, Mahidol University, Bangkok, Thailand; 2 Division of Geriatric Medicine, Department of Medicine, Faculty of Medicine, Chulalongkorn University, Bangkok, Thailand; 3 Geriatric Excellence Center, King Chulalongkorn Memorial Hospital, The Thai Red Cross Society, Bangkok, Thailand; 4 Department of Medicine, Faculty of Medicine, Ramathibodi Hospital, Mahidol University, Bangkok, Thailand; Istanbul University-Cerrahpaşa, Cerrahpaşa Faculty of Medicine, TURKEY

## Abstract

**Background:**

Unintentional weight loss (UWL) is defined as unintentional reduction of more than 5% of baseline body weight over 6 to 12 months. UWL is a common problem in the older adults, resulting in increased rate of morbidity and mortality. With specific reference to Thailand, no information on factors associated with UWL in older adults could be traced. The aims of this research were to identify the factors associated with UWL and to assess the common causes of UWL among older adults in the geriatric outpatient clinic of university hospital.

**Methods:**

A case-control study was conducted from June 1^st^, 2020 to December 31^st^, 2020. Eighty older adults aged 60 years or older were enrolled in the UWL group while the non-UWL group consisted of 160 participants. Data collection was performed by structural questionnaire including baseline characteristics, psychosocial factors, health information, lifestyle behaviors, and medications. The factors associated with UWL were analyzed by using univariate and multivariate logistic regression analysis. Causes of UWL were recorded from electronic medical records.

**Results:**

The mean age of the 240 participants was 79.6 years (SD 7.4). Most patients were female (79.2%) and had fewer than 12 years of education (62.6%). The three common causes of UWL were reduced appetite (20.1%), dementia and behavioral and psychological symptoms of dementia (13.7%) and medications (11.0%). Multivariate logistic regression analysis showed that a Charlson Comorbidity Index (CCI) score of >1 (OR 2.55, 95% CI 1.37–4.73; P = 0.003), vitamin D deficiency (OR 4.01, 95% CI 1.62–9.97; P = 0.003), and hemoglobin level of <12 g/dL (OR 2.47, 95% CI 1.32–4.63; P = 0.005) were factors significantly associated with UWL.

**Conclusions:**

Factors associated with UWL were CCI score >1, vitamin D deficiency, and hemoglobin level of <12 g/dl. The early detection of these associated factors, reduced appetite, dementia and polypharmacy may be important in UWL prevention in older adults.

## Introduction

In recent decades, the aged population has grown rapidly [1]. Worldwide, the number of people 60 years or older in 2017 was estimated at 926 million and is projected to reach 2.1 billion by 2050 [1]. Unintentional weight loss (UWL) is defined as unintentional reduction of more than 5% of baseline body weight within 6 months to a year [2–4]. The population 60 years or older in Thailand has risen markedly from 12 million in 2020 and is predicted to reach 20 million by 2040 [5]. Many older adults live with chronic diseases such as gastrointestinal diseases, dementia, malignancy and depression that require treatment with multiple medications, leading to increase of UWL [6, 7]. The prevalence of UWL among older adults ≥65 years old has been reported as 27% in community dwelling and up to 60% in the nursing-home setting [7]. Besides chronic diseases and their pharmacological treatment, major factors associated with greater risks of UWL are history of hospitalization [6], physical disability [6], psychological problems [8, 9] and age-related physiological changes or physiological anorexia of aging [10] such as the decline of taste perception and olfactory function, decreased saliva production and reduced efficiency of chewing [10–12]. The geriatric population, especially the more vulnerable, has a tendency to experience impaired activity of daily living (ADL) as a result of advancing age and chronic disease, resulting in lack of individual ability in personal preparation and reduced consumption of high-quality food [13–15]. UWL has devastating effects on healthcare infrastructures and health outcomes through the increase of infection [16, 17], bone loss and fracture [18–20], hospitalization [7, 21], poorer quality of life [6, 22, 23], morbidity and mortality [24–26]. However, in the context of Thailand, no previous study has evaluated UWL-associated factors and common causes of UWL among older patients.

The first objective of this study was to explore factors associated with UWL among older patients in the geriatric outpatient clinic of a tertiary care hospital. The second aim was to determine common causes of UWL from medical record reviews among this population.

## Materials and methods

### Study design, setting and participants

The current study was an analysis of a case-control study of 240 patients aged 60 years or older who visited a geriatric outpatient clinic at Ramathibodi Hospital, Mahidol University during the period from June 1, 2020 to December 31, 2020. The participants were classified into two groups: a UWL group and a non-UWL group. Sample size calculation was performed using the n4studies sample size application. Based on the previous data showing poor appetite associated with malnutrition of odds ratio (OR) 2.42 [27], the type I error of 0.01, the type II error of 0.1, exposed proportion of 0.2, and number of controls per case of 2, the sample size to be collected was 240. The UWL group consisted of 80 patients with unintentional loss of at least 5%of baseline body weight over the preceding 12 months. The non-UWL or control group comprised 160 patients without UWL. These patients matched the sex ratio of the UWL group and their age range was no more than 5 years younger or older than that of the UWL subjects. Patients with intentional weight loss, bed-bound status, or no data regarding body weight were excluded from the study. Furthermore, participants were excluded from study if patient or direct relative such as spouse, direct descendant or dependent direct relative in the ascending line does not agree to participate in the study. However, the researchers provided the opportunities for participants or direct relatives to discuss and share ideas for study. Moreover, we answered the questions and provided further information. Finally, if patient or direct relative does not agree, we could not include this patient or direct relative in our study.

### Ethical considerations and consent for participants

This study was approved by the Committee on Human Rights Related to Research Involving Human Subjects, Faculty of Medicine Ramathibodi Hospital, Mahidol University, Protocol Number: COA. MURA2020/991. Participants or their relatives were informed regarding the purpose, the research process and procedure, the advantages and discomforts of the current study. However, they had full right to refuse to participate in the study. All obtained information from participants or direct relatives (spouse, direct descendant or dependent direct relative in the ascending line) was concealed. Then, written informed consent was obtained from participant who had decisional capacity or her direct relative at a geriatric outpatient clinic at Ramathibodi Hospital. All participants were evaluated by informal decisional capacity assessment. The first step is to ensure that participant is able to communicate with assessors, and understand and reasonably process the information in the study. Beyond comprehension, the ability to compare outcomes of research involvement and to conceptualize, and the ability to indicate a logical choice were required as well.

### Data collection and measurement tools

The data were collected from electronic medical records (EMRs) including demographic characteristics: age, sex, marital status; health information factors: comorbidities, psycho-behavioral patterns; current and baseline body weight; current medications: the medication name, doses, frequency, route of administration; healthcare services: history of hospital admission or ER visit within 12 months, medical insurance; laboratory results related UWL: white blood cell count, hemoglobin, blood urea nitrogen, creatinine, serum sodium, serum calcium, serum magnesium, serum phosphorus, aspartate aminotransferase, alanine aminotransferase, alkaline phosphatase, serum albumin levels, blood sugar levels, Thyroid Stimulating Hormone (TSH) levels, total cholesterol levels. Moreover, common causes of UWL among older adults in clinical practice were also determined from EMRs. These common causes of UWL were diagnosed by physicians and were recorded based on the documented International Statistical Classification of Diseases and Related Health Problem 10^th^ Revision (ICD-10) diagnosis.

A structured telephone interview-administered questionnaire was used in this study. The questionnaire comprised information on socio-demographic variables: educational status, lifestyles (drinking, smoking, exercising), income, caregivers, social activity, living situation, feeding behaviors; geriatric conditions: Basic Activity of Daily Living (BADL) [28], Instrumental Activity of Daily Living (IADL) assessments [29], insomnia, falls, chewing and dental problems, visual and auditory impairment [30, 31]; current medications: over-the counter (OTC) medications, supplements.

At 12 months, assessors gathered and recorded all socio-demographic data, health information, lists of all taken medications and laboratory results. Charlson Comorbidity index (CCI) scores [32] were used to predict the one-year mortality for individuals. CCI scores were calculated from comorbidity conditions including cancer, heart disease (a total of 17 conditions). Each condition ranged a score from 1 to 6, depending on the risk of dying related to each one. The summed scores were higher indicating a greater mortality. Functional ability and levels of dependence were assessed by Barthel ADL index [28] and Lawton IADL index [29]. The Barthel ADL index evaluates performances activities including feeding, self-grooming, bathing, toilet use, mobility, dressing, and continence, ranging from a minimum of “0” to a maximum score of “20” with lower scores indicating greater level of incapacity [28]. IADL scales summed the performance on the items of food preparation, housework, shopping, transportation, ability to use telephone and handling medications. The total scores ranged from 0 to 8, with lower scores referring a greater level of dependence [29]. In terms of psychological problems, depression was evaluated by using Patient Health Questionnaire-9 (PHQ-9), ranging from 0 to 27. The high score of PHQ-9 represented a greater level of depressive symptoms, with scores of 5–8 (mild), 10–14 (moderate), and ≥ 15 (severe) [33]. Other socio-demographic data and heath information such as oral and dental health, lifestyle and feeding behaviors, geriatric conditions, and behavioral and psychological symptoms in dementia (BPSD) were evaluated by using a structured telephone interviewer-administered questionnaire. The questionnaire comprised the binary closed question that “Have you had visual impairment in the geriatric condition section?” Participants were informed to report as “Yes” if she or he had visual impairment, and coded as “1”.

### Statistical analysis

All statistical analyses for the current study were performed using the SPSS for Windows Software Package, Version 25.0 (SPSS, Chicago, IL., USA). The descriptive analyses of all characteristic profiles were presented as percentage, mean (standard deviation [SD]) or median (interquartile range [IQR]). The comparative analyses of baseline and clinical characteristics between the UWL and non-UWL groups were performed using Pearson’s chi-square test or Fisher’s exact test for categorical variables and Unpaired Student’s t-test or Mann–Whitney U test for continuous variables. Univariate logistic regression analysis was used first to determine significant variables. The multivariate logistic model was then adjusted for age, sex, history of malignancy, chemotherapy use and significant variables from the univariate logistic model to evaluate independent risk factors for UWL. The results were recorded as odd ratios (ORs) and 95% confidence interval (CI). Statistical significance was valid at the level of p<0.05.

## Results

### Baseline and clinical characteristics

Of the 240 participants, 80 (33.3%) and 160 (66.7%) were categorized as UWL group and non-UWL group respectively. The comparative and descriptive analyses of baseline characteristics of the two groups in the study population are presented in Table 1. The mean age of this population was 79.6 (SD 7.4) years and was not substantially different between the two groups (p = 0.721). The great majority of participants were female (79.2%) and over half of the participants had less than 12 years of education (62.6%). In both groups, less than 5% of the total population were drinkers or smokers. Baseline body weight was not significantly different between the two groups, 55.7 (SD 10.7) kg in the UWL group versus 56.4 (SD 10.1) kg in the non-UWL group (p = 0.194). We found that the number of patients doing less than 30 minutes of exercise per week in the UWL group was substantially higher in comparison with the non-UWL group (p = 0.008). With regard to social status, the number of patients not participating in social activity was significantly greater in the UWL group, compared with the non-UWL group (p = 0.042). By contrast, there was no difference between the two groups in the number of patients living alone, eating alone > 4 days per week, or with inadequate income. In terms of healthcare services, the number of patients with history of hospital admission within 12 months was substantially higher in the UWL group than in the non-UWL group (p = 0.014).

**Table 1:** Baseline characteristics among older patients in the geriatric outpatient clinic.

Characteristics	UWL	Non-UWL	P-value
	(n = 80)	(n = 160)	
	N (%)	N (%)	
Age in years, mean (SD)	79.4 (7.2)	79.7 (7.5)	0.721^#^
Female	63 (78.8)	127 (79.4)	0.911*
Education < 12 years	55 (68.8)	95 (59.4)	0.157*
Single	6 (7.5)	24 (15.0)	0.098*
Baseline body weight (kg), mean (SD)	55.7 (10.7)	56.4 (10.1)	0.194^#^
Current body weight (kg), mean (SD)	50.6 (10.4)	57.1 (10.2)	<0.001^#^
**Lifestyle**			
Drinking	3 (3.8)	5 (3.1)	1.000*
Smoking	2 (2.5)	4 (2.5)	1.000*
Exercise < 30 min per week	53 (66.3)	77 (48.1)	0.008*
**Socioeconomic status**			
Inadequate income	2 (2.5)	4 (2.5)	1.000*
Living alone	6 (7.5)	12 (7.5)	1.000*
Eating alone > 4 days per week	32 (40.0)	65 (40.6)	1.000*
No caregiver	29 (36.3)	70 (43.8)	0.266*
No social activity	53 (66.2)	84 (52.5)	0.042*
**Healthcare services**			
Universal coverage scheme	14 (17.5)	15 (9.4)	0.069*
History of hospital admission within 12 months	18 (22.5)	17 (10.6)	0.014*
History of ER visit within 12 months	19 (23.8)	39 (24.4)	0.915*

**Data are presented as** mean (standard deviation) or n (%).

* Chi-square test,

^#^ Student’s t-test.

**Abbreviations:** SD, standard deviation; kg, kilogram; ER, emergency room.

With regard to comorbidities, participants in the UWL group had significantly more comorbidities than counterparts in the non-UWL group (p = 0.028), as presented in Table 2. Therefore, the number of patients with CCI score >1 in the UWL group was markedly greater compared with the non-UWL group (p = 0.001). In both groups, the participants were more likely to have dyslipidemia, hypertension and osteoporosis. Approximately 43% of this population suffered from dementia. The number of patients with vitamin D deficiency, chronic congestive heart failure and hematologic problems in the UWL group were significantly greater in comparison with the non-UWL group (p<0.05). However, there was marginal difference between the two groups in terms of the number of patients with diabetes mellitus, dementia, behavioral and psychological symptoms of dementia (BPSD) and depression (p>0.05). Regarding geriatric conditions, the majority were more likely to eat orally (98.3%) and use dentures (57.1%), as shown in Table 3. Participants in the UWL group had a greater tendency to suffer falls (>2 episodes) within 12 months in comparison with the non-UWL group (p<0.05). However, the number of participants with oral problems, chewing and swallowing disorders and BADL or IADL impairment were not significantly different between the two groups.

**Table 2:** Clinical characteristics among older patients in the geriatric outpatient clinic.

Characteristics	UWL	Non-UWL	P-value
	(n = 80)	(n = 160)	
	N (%)	N (%)	
**Comorbidities**			
No. of chronic diseases, mean (SD)	5.6 (2.1)	4.9 (2.1)	0.028^#^
CCI, median (IQR)	1.5 (1, 2)	1 (0, 2)	0.005^+^
CCI score >1	40 (50)	46 (28.8)	0.001*
Dyslipidemia	63 (78.8)	128 (80)	0.821*
Hypertension	61 (76.3)	122 (76.3)	1.000*
Osteoporosis	33 (41.3)	68 (42.5)	0.853*
Diabetes mellitus	31 (38.8)	46 (28.8)	0.118*
Hematological problems	12 (15)	11 (6.9)	0.044*
Chronic congestive heart failure	3 (3.8)	1 (0.6)	0.044*
Vitamin D deficiency	14 (17.5)	11 (6.9)	0.011*
Depression	11 (13.8)	20 (12.5)	0.785*
Dementia	37 (46.3)	66 (41.3)	0.168*
BPSD	23 (28.8)	31 (19.4)	0.101*
Parkinson’s disease	2 (2.5)	3 (1.9)	1.000*
Malignancy	13 (16.3)	17 (10.6)	0.214*
Hyperthyroidism	1 (1.3)	2 (1.3)	1.000*
Constipation	4 (5)	14 (8.8)	0.298*
COPD	6 (7.5)	4 (2.5)	0.088*

**Data are presented as** mean (standard deviation), n (%), or median (interquartile range).

* Chi-square test,

^#^ Student’s t-test,

^+^ Mann–Whitney U test.

**Abbreviations:** SD, standard deviation; CCI, Charlson Comorbidity Index; IQR, interquartile range; BPSD, behavioral and psychological symptoms of dementia; COPD, chronic obstructive pulmonary disease.

**Table 3:** Geriatric conditions among older patients in the geriatric outpatient clinic.

Characteristics	UWL	Non-UWL	P-value
	(n = 80)	(n = 160)	
	N (%)	N (%)	
**Geriatric conditions**			
BADLs, mean (SD)	17.9 (3.0)	18.1 (3.9)	0.734^#^
BADL impairment	4 (5.0)	11 (6.9)	0.572*
IADLs, mean (SD)	6.0 (2.9)	6.4 (3.0)	0.252^#^
IADL impairment	41 (51.3)	65 (40.6)	0.118*
Insomnia	7 (8.8)	13 (8.1)	0.869*
History of fall > 2 episodes in previous 12 months	15 (18.8)	15 (9.4)	0.038*
Visual impairment	18 (22.5)	42 (26.3)	0.510*
Hearing impairment	19 (23.8)	67 (41.9)	0.006*
Chewing problems	25 (31.3)	54 (33.8)	0.698*
Denture use	43 (53.8)	94 (58.8)	0.461*
Denture problems	28 (35.0)	51 (31.9)	0.731*
Oral feeding	78 (97.5)	158 (98.8)	0.602*

**Data are presented as** mean (standard deviation) or n (%).

* Chi-square test,

^#^ Student’s t-test.

**Abbreviations:** SD, standard deviation; BADLs, basic activities of daily living; IADLs, instrumental activities of daily living.

### Prescribed medication use

The number of prescribed medications ranged from 1 to 20. The mean of number of prescribed medications was 8.9 (SD 3.6) in the UWL group and 8.7 (SD 3.9) in the non-UWL group, as presented in Table 4. Most participants (87.1%) consumed at least five medications (polypharmacy). There was no difference in the number of patients exposed to polypharmacy between the two groups (P >0.05). The three most frequently taken medications were statins (78.3%), followed by calcium channel blockers (50.4%) and calcium (49.6%). In the UWL group, the prescription of chemotherapy and Dipeptidyl Peptidase-4 (DPP-4) Inhibitor was significantly higher in comparison with the non-UWL group. On the contrary, the number of prescribed medications in metformin, antidepressants and acetylcholinesterase inhibitors (AChEIs) were marginally different between the two groups.

**Table 4:** Prescribed medication use among older patients in the geriatric outpatient clinic.

Characteristics	UWL	Non-UWL	P-value
	(n = 80)	(n = 160)	
	N (%)	N (%)	
No. of medications, mean (SD)	8.9 (3.6)	8.7 (3.9)	0.695^#^
Polypharmacy (≥ 5 drugs)	71 (88.8)	138 (86.3)	0.586*
Statins	61 (76.3)	127 (79.4)	0.580*
Calcium channel blockers	37 (46.3)	84 (52.5)	0.361*
Calcium	37 (46.3)	82 (51.3)	0.465*
AChEIs	27 (33.8)	58 (36.3)	0.703*
Antidepressants	27 (33.8)	54 (33.8)	1.000*
Metformin	20 (25.0)	27 (16.9)	0.135*
Beta-blockers	21 (26.3)	43 (26.9)	0.918*
DPP-4 inhibitors	18 (22.5)	19 (11.9)	0.032*
Chemotherapy	4 (5)	1 (0.6)	0.044*
Iron	10 (12.5)	11 (6.9)	0.146*
Antipsychotics	9 (11.3)	17 (10.6)	0.883*
ACEIs	9 (11.3)	13 (8.1)	0.429*
Anticholinergics	7 (8.8)	10 (6.3)	0.477*
Benzodiazepines	8 (10)	25 (15.6)	0.233*
Bisphosphonates	12 (15)	18 (11.3)	0.408*
Digitalis	1 (1.3)	0 (0)	0.333*
Antiepileptics	7 (8.8)	14 (8.8)	1.000*
Laxatives	17 (21.3)	23 (14.4)	0.178*

**Data are presented as** mean (standard deviation) or n (%).

* Chi-square test,

^#^ Student’s t-test.

**Abbreviations:** SD, standard deviation; AChEIs, acetylcholinesterase inhibitors; DPP-4, Dipeptidyl Peptidase-4; ACEIs, angiotensin-converting enzyme inhibitors.

### Laboratory results

The mean hematocrit level in the UWL group was significantly lower than that in the non-UWL group (p = 0.002), as shown in Table 5. The albumin, cholesterol and glucose levels showed no difference between the two groups.

**Table 5:** Laboratory results among older patients in the geriatric outpatient clinic.

Characteristics	UWL	Non-UWL	P-value
	(n = 80)	(n = 160)	
White blood cell count (cells/mm^3^), mean (SD)	6,543.7 (2,060.6)	6,865.0 (2,413.4)	0.326^#^
Hemoglobin (g/dL), mean (SD)	12.1 (1.5)	12.7 (1.4)	0.002^#^
Blood urea nitrogen (mg/dL), mean (SD)	16.4 (6.2)	15.5 (7.7)	0.383^#^
Creatinine (mg/dL), mean (SD)	0.9 (0.3)	0.9 (0.4)	0.852^#^
Sodium (mmol/L), mean (SD)	140.9 (2.4)	140.8 (2.8)	0.893^#^
Calcium (mg/dL), mean (SD)	9.2 (0.5)	9.2 (0.5)	1.000^#^
Magnesium (mg/dL), mean (SD)	2 (0.2)	2 (0.2)	0.840^#^
Phosphate (mg/dL), mean (SD)	3.6 (0.6)	3.5 (0.6)	0.415^#^
Aspartate aminotransferase (U/L), mean (SD)	30.2 (13.2)	28.7 (9.2)	0.346^#^
Alanine aminotransferase (U/L), median (IQR)	17 (13, 27)	20 (15, 27)	0.411^+^
Alkaline phosphatase (U/L), median (IQR)	102 (94, 116)	80 (66, 100)	0.095^+^
Albumin (g/L), mean (SD)	38 (4)	38.4 (3.1)	0.326^#^
Blood sugar (mg/dL), mean (SD)	106.5 (29.2)	109.9 (32.1)	0.457^#^
Thyroid Stimulating Hormone (uIU/mL), median (IQR)	1.5 (1.1, 2.6)	1.6 (1.3, 2.8)	0.576^+^
Total cholesterol (mg/dL), mean (SD)	172.9 (37.4)	177.5 (35.7)	0.372^#^

**Data are presented as** mean (standard deviation) or median (interquartile range).

^#^ Student’s t-test,

^+^ Mann–Whitney U test.

**Abbreviations:** mm, millimeter; g, gram; dL, deciliter; mg, milligram; mL, milliliter; mmol, millimole; L, liter; U, unit; uIU, micro international unit; SD, standard deviation; IQR, interquartile range.

### Factors associated with unintentional weight loss

The significant variables for UWL after univariate logistic regression analysis are presented in Table 6. These remained significant variables, sex, age, history of malignancy, and chemotherapy use were further adjusted in the process of multivariate logistic regression analysis. After multivariate adjustment, the independent risk factors for UWL were CCI score >1 (OR 2.55, 95% CI 1.37–4.73; P = 0.003), vitamin D deficiency (OR 4.01, 95% CI 1.62–9.97; P = 0.003), and hemoglobin level of <12 g/dL (OR 2.47, 95% CI 1.32–4.63; P = 0.005) However, none of the specific prescribed medication was associated with UWL after adjusted analyses.

**Table 6:** Univariate and multivariate analysis of factors associated with UWL.

The associated factors	Univariate		Multivariate^a^	
	OR (95%CI)	p-value	OR (95%CI)	p-value
Hematologic problems	2.39 (1.01–5.69)	0.049		
Exercise <30 min/week	2.11 (1.21–3.69)	0.008*		
Vitamin D deficiency	2.87 (1.24–6.67)	0.014	4.01 (1.62–9.97)	0.003*
CCI score >1	2.48 (1.42–4.32)	0.001*	2.55 (1.37–4.73)	0.003*
History of hospital admission within 12 months	2.44 (1.18–5.05)	0.016		
No social activity	1.77 (1.02–3.10)	0.044		
DPP 4 inhibitors	2.15 (1.06–4.38)	0.034		
Hemoglobin level of <12 g/dL	2.12 (1.22–3.99)	0.008*	2.47 (1.32–4.63)	0.005*
History of fall >2 episodes in previous 12 months	2.23 (1.03–4.83)	0.042		

^a^Adjusted for age, sex, history of malignancy, chemotherapy use and significant variable from unadjusted model,

*P<0.01.

**Data are presented as** odds ratio (95% confidence interval).

**Abbreviations:** CCI, Charlson Comorbidity Index; DPP-4, Dipeptidyl Peptidase-4; min, minute; g, gram; dL, deciliter; OR, odds ratio; CI, confidence interval.

### Common causes of UWL in clinical practice

The causes of UWL were reported in the EMRs. The three most common causes of UWL were reduced appetite (20.2%), followed by dementia with BPSD (13.8%) and medications (10.1%).

## Discussion

The present study has reported the factors associated with UWL and has explored the common causes of UWL in clinical practice among older patients visiting a geriatric outpatient clinic during the period from June 1, 2020 to December 31, 2020.

In terms of baseline characteristics, analyses of preceding studies [6, 34] presented a positive relationship between UWL and reduced social activity among older adults which is in accordance with our current study. With regard to comorbidities, the presence of more comorbidities is associated with greater risk of UWL, which is in line with data from previous studies [6, 35]. The most common chronic diseases among patients with UWL are dyslipidemia, followed by hypertension and osteoporosis. The number of patients with vitamin D deficiency, chronic congestive heart failure and hematological problems in the UWL group were significantly higher, comparing with the non-UWL group. According to these studies [36–40], patients with congestive heart failure had a tendency to have UWL, resulting from hepatic congestion, reduced motility of gastrointestinal tract, increased work of breathing and weakness. Nevertheless, a relationship between chronic diseases including dementia, depression, and UWL was not found in the present study, in contrast to previous studies [34, 41]. In terms of geriatric conditions, older patients with a history of falls were substantially more numerous in the group with UWL [6]. According to the study of Fougère, weight loss resulted in loss of muscle lean mass which was a major cause of sarcopenia [42]. This condition was associated with loss of the muscle strength and increased falls [42]. Other independent predictors of UWL were oral disorders and chewing and swallowing problems [6] although this was not apparent in the current study.

Older patients are prone to simultaneously use five or more medications to treat various chronic diseases. In Thailand, approximately 70% of older adults in outpatient clinics experience to polypharmacy [43]. According to recent studies [2–4, 44, 45], polypharmacy was associated with UWL, which contradicts the findings of our current study. Amongst UWL patients, the most common prescribed medications were statins, calcium channel blockers and calcium, which were prescribed for treatment of common co-morbidities including dyslipidemia and hypertension. The prescription of chemotherapy was significantly higher in older patients with UWL, similar to previous studies [46, 47]. Chemotherapy contributes to changes in taste such as altered structure of taste buds and number of taste cell receptors, and detection of drug sensation via saliva [48, 49]. These alterations in flavor perception have negative impacts on nutritional status and body weight [50–52]. Furthermore, chemotherapy induces gastrointestinal side effects including nausea and vomiting. In contrast to our results, other studies have shown a relationship between UWL and the prescription of metformin, antidepressants and AChEIs [53]. Upon univariate logistic regression analysis, we found that ADL, cognitive status and specific medications were not related to an increase of UWL, which contrasts to data from recent studies [6, 54, 55].

After multivariate logistic regression analysis, the predictors of UWL were revealed to be CCI score >1, vitamin D deficiency, and hemoglobin level of <12 g/dL. As would be expected, UWL was instead associated with complex comorbidity [7]. Both increased number of comorbidities and experiencing a flare-up of chronic disease are also potential factors contributing to UWL [6, 35]. In several studies, an inadequate vitamin 25(OH)D status was reported to contribute to reduced muscle mass, leading to weight loss [56–58]. The relevant mechanism of vitamin 25(OH)D status and muscle mass is explained by the vitamin D receptor in skeletal muscle which plays a major role in the process of muscle protein turnover [59]. The stimulation of vitamin D receptor with adequate vitamin D may activate the synthesis of muscle protein [59, 60] and may decrease muscle fiber atrophy [61]. Reduced hemoglobin or anemia may be the cause of frailty and weight loss [62] because anemia may diminish tissue oxygenation, contributing to decreased muscle synthesis and impaired muscle strength [63]. Conversely, malnutrition or weight loss results in many nutritional deficiency diseases such as the insufficiency of vitamin D, vitamin B12 and iron, leading to anemia and vitamin D deficiency [64].

The most common causes of UWL in clinical practice revealed by the current study were poor appetite [6], dementia with BPSD [65] and medications. Unlike preceding reports, the three most common causes of UWL were gastrointestinal disorders, followed by malignant diseases and psychological problems [34, 66, 67]. Self-reported reduced appetite was significantly more frequent in older patients with UWL in the studies by Sorbye et al. and Mowé et al. [6, 68]. In terms of dementia, especially in Alzheimer’s disease, the degenerative process of neurons of the olfactory epithelium and reduced olfactory sensitivity are present throughout neurodegeneration, leading to decreased appetite and food intake [41]. Furthermore, patients with Alzheimer’s disease are more likely to have the increase of resting energy expenditure, impaired eating ability, and chewing and swallowing disorders [69–72]. Therefore, it is not beyond expectation that dementia is associated with UWL [6]. In the context of drug-induced weight loss, many medications play a causative role in UWL by various mechanisms including anorexia (metformin, digoxin), dry mouth (anticholinergics, loop diuretics), dysphagia (chemotherapy) and nausea or vomiting (metronidazole, iron) [3, 34, 73].

A comprehensive history review and physical examination for potentially medical, psycho-behavioral, socioeconomic, medication and intake-related issues should be completely evaluated in older patients with UWL to identify all associated causes of UWL. Besides history taking and physical examination, laboratory results could further guide areas of concern to reach an accurate diagnosis.

### The strengths and limitations

To our knowledge, no previous study has explored factors associated with UWL among older adults in the context of a university hospital in Thailand. The strength of the current study includes the comprehensive data collected from several domains using a combination of medical record reviews with telephone interviews. However, several limitations have to be taken into consideration. First, this study enrolled only older adults visiting a geriatric outpatient clinic and those with bed-bound status were excluded. Therefore, the results may not be applicable to all older patients. Second, a cause-and-effect association between UWL and variable factors could not be assumed. Future longitudinal studies should be performed to address this limitation. Third, when conducting telephone interviews, recall bias may have taken place because some participants, including demented patients, who were unable to retrieve accurate information. Finally, unmeasured confounding factors may remain, although adjusted multivariate logistic regression analysis was rigorously performed.

### Further research and implications

Further research would be better to do study in multicenter and multilevel of care settings to provide generalization. The intervention or clinical practice guideline for UWL prevention including tools or criteria should be developed to early detect patients at risk of UWL. Moreover, the result findings are useful for understanding the factors associated with UWL in this vulnerable population and provide important information to assist physicians in the design of comprehensive geriatric assessment to identify UWL in a high-risk population.

## Conclusion

The most common causes of UWL in clinical practice were reduced appetite, dementia with BPSD and medications. The independent factors associated with UWL were Vitamin D deficiency, CCI score >1, and hemoglobin level of <12 g/dL. Therefore, the early detection of these associated factors, reduced appetite, dementia and polypharmacy may be important for the prevention of UWL in older adults. Furthermore, the useful results from the current study could be used for development of comprehensive assessment tools to identify and early detect UWL in older adults as a high-risk population in the future.

## Supporting information

S1 File Causes of unintentional weight loss from electronic medical records. (DOC)
