# Supplementary material for: Factors associated with unintentional weight loss among older adults in a geriatric outpatient clinic of university hospital
Source: PLoS One. 2021 Nov 18;16(11):e0260233. doi: 10.1371/journal.pone.0260233 (PMC8601429; doi:10.1371/journal.pone.0260233)
Supplement: S1 File — (DOC) [file pone.0260233.s001.doc]

**S1 File. Causes of unintentional weight loss from electronic medical records**

| **Causes of UWL from electronic medical records** | **(n=109)**  **N (%)** |
| --- | --- |
| Reduced appetite | 22 (20.2) |
| Dementia with BPSD | 15 (13.8) |
| Medications | 11 (10.1) |
| Malignancy | 10 (9.2) |
| Unexplained unintentional weight loss | 10 (9.2) |
| Mood disorders | 10 (9.2) |
| Increased physical activity | 6 (5.5) |
| Chewing problems | 5 (4.6) |
| GI problems | 5 (4.6) |
| Medical illness | 4 (3.7) |
| Social problems | 4 (3.7) |
| Low physical activity | 3 (2.8) |
| Restrict calories | 3 (2.8) |
| Swallowing problems | 1(0.9) |
| **Total** | 109 (100) |

**Data are presented as** n (%).

**Abbreviations:** UWL, unintentional weight loss; BPSD, behavioral and psychological symptoms of dementia; GI, gastrointestinal
